# Service Attributes and Acceptability of Digital and Nondigital Depression Management Methods Among Individuals With Depressive Symptoms: Survey Study

**DOI:** 10.2196/55450

**Published:** 2024-12-19

**Authors:** Larry Auyeung, Winnie W S Mak, Ella Zoe Tsang

**Affiliations:** 1 Department of Psychology The Chinese University of Hong Kong Hong Kong SAR China (Hong Kong)

**Keywords:** eHealth, acceptability, user preference, diffusion of innovation, mental health services

## Abstract

**Background:**

Academic research on digital mental health tends to focus on its efficacy and effectiveness, with much less attention paid to user preferences and experiences in real-world settings.

**Objective:**

This study aims to analyze service characteristics that service users value and compare the extent to which various digital and nondigital mental health treatments and management methods fulfill users’ expectations.

**Methods:**

A total of 114 people with at least moderate levels of depressive symptoms (as measured by Patient Health Questionnaire–9 score ≥10) completed a web-based questionnaire measuring their awareness and adoption of digital mental health services and their valuation of 15 psychological service attributes, including effectiveness, credibility, waiting time, and more. They were also assessed on their expectations toward seven common mental health treatments and management methods, including (1) face-to-face psychological intervention, (2) medication, (3) guided internet-based psychological intervention, (4) face-to-face counseling service, (5) self-guided mental health apps for depression, (6) self-help bibliotherapy, and (7) psychological intervention via videoconferencing.

**Results:**

A Friedman test with a Dunn posttest showed the average importance rank of “effectiveness” was significantly higher than all other measured attributes. “Privacy,” “credibility,” and “cost” were ranked as equally important. Participants rated face-to-face psychological intervention the most effective management method, while other digital management methods were perceived as less effective. Medication was perceived as the least appealing method, while other methods were deemed equally appealing. Face-to-face psychological intervention, medication, and counseling were considered less satisfactory due to their higher costs and longer waiting times when compared to digital services. Repeated measures ANOVA showed some forms of management method were more likely to be adopted, including guided internet-based psychological intervention, psychological intervention via videoconferencing, face-to-face psychological intervention, and face-to-face counseling services provided by a counselor as compared to self-guided mobile apps, self-help bibliotherapy, and medication.

**Conclusions:**

The study highlights the importance of considering multiple service attributes beyond effectiveness in depression management methods, despite effectiveness being regarded as the most crucial factor using the rank method. Compared to nondigital services, digital services were identified as having specific strengths as perceived by users. Future dissemination and promotion efforts may focus on debunking myths of guided internet-based psychological intervention as a less effective option and promoting the particular service strengths of digital services.

## Introduction

### Depression and the Emergence of Digital Mental Health

Depressive disorders are highly prevalent and have a substantial impact on disability, mortality rates, productivity decline, and health care expenses [1]. Psychological interventions have been proven to be effective in treating depression [2]. They are widely regarded as the first-line treatment for major depression [2,3] and are the preferred choice of treatment for most individuals with depression [4,5]. Despite the proven effectiveness and preference [6] for psychological interventions, there is a low use rate among individuals with depression [7]. This can be attributed to several factors, including the time commitment, the need to travel, the high cost, and waiting times [8-10].

One potential solution to overcome these barriers is to offer digital mental health service options, such as internet-based cognitive behavioral therapy and mobile apps [11]. It is worth noting that some of the digital mental health services (eg, internet-based cognitive behavioral therapy) have demonstrated comparable effectiveness to face-to-face interventions [12]. The advancement of digital treatment options is speculated to be one of the reasons for the increased proportion of individuals receiving psychological interventions for depression [9]. In the past, digital mental health services were often limited to psychological intervention via videoconferencing, which requires therapists and users to conduct sessions in real time through video calls. This synchronous approach can be less flexible due to the need for scheduled appointments. In recent decades, internet-based psychological interventions have evolved and become more sophisticatedly investigated, providing structured online programs with regular support and guidance, thereby offering users greater flexibility. In the United Kingdom, certain internet-based psychological interventions for mental health conditions have been endorsed and recommended in the clinical treatment guidelines [13]. Amid the COVID-19 pandemic, there has been a notable increase in the adoption of internet-based psychological intervention. Particularly, there has been a remarkable 5-fold surge in monthly registrations for courses that specifically address depression and anxiety symptoms [14]. In Hong Kong, although the adoption and use of digital mental health services has been much slower compared to western countries, digital mental health services have already been introduced independently outside of the formal health care system by local universities and academic institutions [15,16] As efforts to establish and expand digital mental health services continue, it is crucial to assess society’s readiness to embrace this innovative service. Understanding the public’s perception of these novel depression-focused mental health services is essential for identifying the factors that drive acceptance and potential implementation barriers. This assessment also ensures preparedness and legitimacy for broader implementation [17].

### The Importance of Users’ Views and Acceptance of Digital Mental Health Services

When implementing digital mental health services, it is important to consider users’ opinions and acceptance. By using the Unified Theory of Acceptance and Use of Technology [18] and relevant research [19], acceptability in this study refers to (1) how users perceive different attributes of the service (such as effectiveness, credibility, and waiting time [20,21]) and (2) their intention to use digital mental health treatment services [22]. The importance of considering acceptability has recently gained much recognition in the implementation of new health care interventions [23]. Treatment acceptability is also seen as a crucial factor for the successful dissemination and implementation of new health services. Because a treatment could be clinically effective, its adoption and use may be compromised if it is unacceptable to users, resulting in an implementation gap [24,25].

Despite the importance of users’ views, there are several significant research gaps. First, in the field of treatments for depression, studies investigating treatment acceptability have predominantly focused on traditional face-to-face psychological interventions and pharmacological therapy, with few studies focused on digital mental health services [26,27]. Among the few, preference for digital mental health services has been shown to tend to be lower than face-to-face options, and the proportion of those who preferred digital mental health services [19,28] varied wide from 25.6% to 93% [29]. Although studies reported that a substantial amount of people indicate a willingness to use digital intervention, face-to-face psychological interventions still appeared to be the more preferred option [30]. Thus, research is needed to ascertain why people with depressive symptoms maintained a preference for in-person psychological interventions as opposed to the equally effective [31] and yet cheaper options in digital form [32]. Second, to the best of our knowledge, most of the studies that take user perspectives into account have used “general” measures of acceptance [30], No study has attempted to understand how different digital and nondigital mental health services or management methods meet expectations of individuals with depression regarding “specific” service aspects, including perceived effectiveness, cost, privacy, credibility, and more. Finally, most of the current evidence on acceptability was based on samples without depressive symptoms above the clinical threshold [19,28], which limits the validity of the conclusions drawn as participants had to imagine whether they would use the services if they were undergoing a depressive episode, which could be cognitively demanding.

In fact, the integration of digital mental health services into health care systems is no small investment, especially when advanced techniques in computational and data science are increasingly incorporated in the development of digital mental health services [33]. It is thus worthwhile to study which service attributes people value the most (eg, cost, effectiveness, and waiting time) and how digital mental health services might meet or might not meet their expectations. It is also important to update the dissemination progress of digital mental health services by studying users’ awareness and likelihood of using digital mental health services. Understanding service strengths and weaknesses from users’ perspectives could also inform our direction in direct-to-consumer marketing or social campaigns [34,35] that aim at increasing the market share of digital mental health services by introducing traditional service preferers to use digital mental health services.

### Study Design and Objectives

This study aimed to investigate service users’ acceptability between traditional and emerging digital mental health services in Hong Kong, a major Asian city that has much need and resources to implement and scale up digital mental health services and can be an important reference for other Asian cities to follow suit.

This study adopted a web-based cross-sectional survey. It had three objectives: (1) to investigate awareness and adoption of digital mental health services and to identify valued attributes for depression management; (2) to explore how digital mental health services compare to other service options for depression in fulfilling users’ expectations regarding service attributes, such as effectiveness, cost, credibility, and more; and (3) to compare the likelihood of using different management methods for depression.

## Methods

### Participants

A community sample of 114 adults was recruited through promotion authors’ institution mass mail, social media, flyers, posters, and web advertisements. They met the eligibility criteria of (1) being aged ≥18 years, (2) being Chinese speaking, and (3) having a Patient Health Questionnaire (PHQ)–9 [36] result ≥10 (a cutoff point of 10 on the PHQ-9 has a pooled sensitivity of 0.78 and a pooled specificity of 0.87 for detecting major depressive disorder [37]). Recruitment took place in both community and internet-based settings. Participants self-enrolled in the study by scanning the QR code on the study flyers or by clicking the study website link, and they independently completed an anonymous series of questionnaires after providing informed consent. No initial contact with the potential participants was made.

### Ethical Considerations

The study was approved by the Survey and Behavioral Research Ethics Committee of the authors’ institution (SBRE-21-0326). Participants voluntarily provided informed consent to participate in the study and were assured of their right to withdraw at any time without consequence. Study data were protected by a password on the computer, and only the research team had access to it. Participants were offered HK $50 (US $6.42) as a token of thanks upon completion of the survey.

### Measures

All the measures were collected via a web-based questionnaire platform, Qualtrics (Qualtrics International Inc). Participants completed the survey at their convenience upon receiving the access link through our promotional channels mentioned above.

#### Demographics

Sociodemographic characteristics, including gender, age, education level, perceived socioeconomic status, and employment status, were obtained. Depression severities were measured by the PHQ-9 [36], which provides a brief 9-item measure of current depression symptoms using a 4-point Likert scale ranging from 0 (not at all) to 3 (nearly every day). The PHQ-9 categorizes depression severity with scores of 10 to 14 as moderate, 15 to 19 as moderately severe, and 20 to 27 as severe [36].

#### Awareness and Adoption of Digital Mental Health Services

The scale started with “Over the past ten years, there has been a surge of development in digital health service, people who experience depressive symptoms may choose to seek help from these services.” To assess the level of awareness and adoption of digital mental health treatment services, participants were asked to indicate whether they had heard of, previously tried, or were currently using guided internet-based psychological intervention, mental health apps for depression, and psychological intervention via videoconferencing. Binary responses were used to gather this information (yes=1 and no=0).

### Importance of 15 Psychological Service Attributes

As revised in the study by Musiat et al [19], the scale used in this study included 15 domains or qualities of mental health treatments that may influence individuals’ decisions to engage in a particular treatment option for depression. These domains were (1) effectiveness, (2) privacy protection, (3) credibility, (4) low or no cost, (5) personalization, (6) accessibility with little or no waiting time, (7) anonymity of access, (8) provision of timely support, (9) feedback provision, (10) convenience of access time, (11) appeal, (12) absence of side effects, (13) ability to motivate service users, (14) ability to monitor mental health status, and (15) low or no transportation cost. Participants were asked to rate the importance of each of these 15 attributes on a 7-point rating scale ranging from 1 (“not important at all”) to 7 (“very important”). Participants were instructed to consider mental health treatments in general and to indicate what they would consider important if they were seeking help at the present time. Given literature suggests that absolute rating using Likert-type scales has some inherent disadvantages, such as response styles, in which respondents may demonstrate systematic tendencies in their choices of certain response options [38]. For example, respondents may be inclined to provide extreme responses and rate all service attributes as extremely important, which prevents the respondents’ true characteristics from being obtained [39,40]. Thus, a simple ranking exercise was followed; participants were asked to rank the importance of the psychological service attributes from 1 to 15. Participants were asked to rank the 15 attributes in order of importance, from 1 (“most important”) to 15 (“least important”) if they were currently seeking help.

### Expectation Fulfillment Regarding 7 Common Mental Health Treatments or Management Methods

To investigate participants’ expectations toward common mental health treatments and management methods, they were asked to rate to what extent they believed each of the 15 domains from the previous scale would be met by 7 different treatment and management options for depression. These options included (1) traditional face-to-face psychological intervention provided by a clinical psychologist, (2) psychotropic medication, (3) guided internet-based psychological intervention, (4) counseling service provided by a counselor, (5) mobile app for depression, (6) self-help literature, and (7) psychological intervention via videoconferencing. Expectation ratings were obtained on a 7-point rating scale, ranging from 1 (“would not meet my expectations at all”) to 7 (“would fully meet my expectations”).

### Likelihood of Use

To assess the likelihood of using each of the 7 common mental health treatments and management methods, participants were asked to rate on a scale of 1 (“very not likely”) to 7 (“very likely”) how likely they would be to use each treatment and management method mentioned earlier.

### Statistical Analysis

Statistical analyses were performed using SPSS (version 26.0; IBM Corp) [41] and R software (version 4.1.0; R Foundation). The difference in the proportion of participants who were aware of or adopted digital mental health services was analyzed using the Cochran Q test. Differences in the importance of psychological service attributes, using an absolute rating scale, were analyzed using repeated measures ANOVA. In terms of the ranked ratings of the importance of psychological service attributes, the Friedman test with Dunn posttest, a nonparametric statistical test for analyzing repeated measures data, was used. The *fmsb* package was used to generate radar plots of expectations fulfillment of 7 different treatment and management options for depression [42]. The difference in likelihood of use among 7 depression management methods was analyzed using repeated measures ANOVA with Bonferroni adjustment for multiple comparisons in the post hoc test. The level of statistical significance was set at *P*<.05. Less than 5% missing data were observed, and complete case analyses were used.

## Results

### Participants and Demographics

A total of 86 participants were excluded for having PHQ-9 scores <10. 114 eligible participants had a mean age of 26.8 (SD 6.35) years, of whom 77.2% (88/114) were women. The majority (73/114, 64%) had no prior diagnosis of mental disorders, while 59.6% (68/114) reported at least moderately severe depressive symptoms, as indicated by a PHQ-9 score of ≥15.

### For Objective 1: To Investigate Awareness and Adoption of Digital Mental Health Services and to Identify Valued Attributes for Depression Management

#### Awareness and Adoption

In total, 42.1% (48/114) of participants had previously tried psychological intervention, and 25.4% (29/114) had received psychotropic medication. Regarding awareness and adoption of digital mental health services, most of the participants were aware of the existence of guided internet-based psychological intervention, mobile app for depression, and psychological intervention via videoconferencing, with 57.5% (65/113) to 77% (87/113) of the participants having heard of the mental health services mentioned earlier (Table 1). A related-samples Cochran Q test showed that the awareness of guided internet-based psychological intervention, a mobile app for depression, and psychological intervention via videoconferencing was significantly different, *χ*^2^_2_=21.0, *P*<.001, a pairwise comparison indicated that more participants were aware of the presence of guided internet-based psychological intervention, followed by psychological intervention via videoconferencing and a mobile app for depression. As for the experience and the current adoption of the 3 digital mental health services, only 12.6% (14/111) to 19.5% (22/113) of the participants have ever used digital mental health treatment services, and only 3.6% (4/111) to 6.3% (7/112) of the participants were currently using digital mental health treatment services. The Cochran Q test did not indicate any differences among the 3 proportions, *χ*^2^_2_=2.7, *P*>.26; *χ*^2^_2_=1.4, *P*>.497.

**Table 1:** Awareness and adoption of digital mental health treatment services.

	Aware of, n/N (%)	Tried, n/N (%)	Currently using, n/N (%)
Guided internet-based psychological intervention	87/113 (77)	22/113 (19.5)	6/112 (5.4)
Mobile app for depression	65/113 (57.5)	18/113 (15.9)	4/111 (3.6)
Psychological intervention via videoconferencing	74/111 (66.1)	14/111 (12.6)	7/112 (6.3)

#### Importance of 15 Psychological Service Attributes

#### Absolute Importance via Rating

Participants rated all 15 investigated attributes as important, with high mean scores ranging from 5.3 to 6.4 and low SDs ranging from 1.0 to 1.8 (Table 2). Thus, the descriptive statistics indicated that people with depressive symptoms highly valued all the positive characteristics of mental health services. However, using repeated measures ANOVA, some attributes were more highly rated than others: *F*_14,95_=11.00, *P*<.001, *η*_partial_^2^=0.62. A pairwise comparison showed that the attribute “privacy” was significantly more highly valued than all other attributes expect side effects. Notedly, the pairwise comparison showed that people with depressive symptoms valued a large bundle of service attributes as equally important, with no significant differences in their absolute importance. These service attributes include feedback provision, anonymity, side effects, personalization, waiting time, flexible time in service provision, effectiveness, credibility, being able to help users to monitor their mental health status, and timely support.

**Table 2:** Absolute importance of different attributes of psychological services.

Attribute	Order by mean importance	Importance, mean (SD)
Privacy	1	6.4 (1)
Feedback provision	2	6.1 (1)
Anonymity	3	6.1 (1.1)
Side effects	4	6 (1.2)
Personalization	5	6 (1.1)
Cost	6	6 (1.3)
Flexible time in service provision	7	5.9 (1.2)
Effectiveness	8	5.9 (1.2)
Credibility	9	5.8 (1.2)
Help users to monitor their mental health	10	5.8 (1.2)
Timely support	11	5.8 (1.7)
Being able to motivate service users	12	5.7 (1.2)
Waiting time	13	5.7 (1.4)
Transportation cost	14	5.4 (1.8)
Appealing	15	5.3 (1.3)

#### Relative Importance via Ranking

“Effectiveness” was on average ranked as the most important service attribute in a comparative sense, with more than two-thirds of the participants (84/113, 74.3%) ranked it as the top 3 most important attribute in psychological service. Following “effectiveness,” it comes with “privacy,” “credibility,” and “cost” having the highest mean rank of importance (Table 3), with more than one-third of the participants ranked one of the attributes mentioned here as the top 3 most important attributes in psychological service.

**Table 3:** Ranked importance of different attributes of psychological services.

Attributes	Rank, mean (SD)
Effectiveness	2.8 (2.5)
Privacy	5.2 (3.3)
Credibility	5.4 (3.4)
Cost	5.9 (3.8)
Personalization	8.1 (3.2)
Waiting time	8.2 (3.5)
Timely support	8.5 (4.4)
Anonymity	8.6 (5.5)
Feedback provision	8.9 (3.6)
Flexible time in service provision	8.9 (3.3)
Appealing	9.2 (4.2)
Side effects	9.3 (4.4)
Being able to motivate service users	9.4 (3.3)
Help users to self-monitor their mental health	10.5 (4)
Transportation cost	11 (3.3)

A Friedman test was conducted to determine if the differential mean ranks had inferential value. The result indicated that some of the surveyed attributes were ranked substantially higher, *χ*^2^_4_=387, *P*<.001. A post hoc pairwise Dunn test of the rank-ordered importance showed that the average importance rank of “effectiveness” significantly differed from all other measured attributes. Yet, it should be noted that the average importance rank of “privacy,” “credibility,” and “cost” was not statistically different (*P*>.99). People with depressive symptoms ranked these 3 attributes, “privacy,” “credibility,” and “cost,” as equally important, following “effectiveness.” The average importance ranks of “personalization,” “waiting time,” “timely support,” “anonymity,” “feedback provision,” “flexible time in service provision,” “appealing,” and “potential side effects,” were not statistically different. Finally, the attributes “help users to self-monitor their mental health status” and “transportation cost” were ranked significantly lower in importance as compared to each of the top 6 attributes that participants ranked as important, all *P*<.02.

### For Objective 2: To Explore How Digital Mental Health Services Compared to Other Service Options for Depression in Fulfilling Users' Expectations

#### Effectiveness, Credibility, and Appeal

Regarding effectiveness, a significant difference was found between treatments and management methods (*F*_6,104_=17.5, *P*<.001, partial *η*^2^=0.50). Participants rated face-to-face psychological intervention as the most effective treatment and management method for depression, while guided internet-based psychological intervention, mobile apps, psychological intervention via videoconferencing, and counseling were perceived as less effective compared to face-to-face psychological intervention. Medication and bibliotherapy or self-help books were rated as the least effective.

For credibility, a significant difference was found between treatments and management methods (*F*_6,104_=24.78, *P*<.001, partial *η*^2^=0.59). Face-to-face psychological intervention was rated as the most credible method for depression management, while guided internet-based psychological intervention, counseling, and medication were perceived as less credible but to a similar extent. Mobile apps and bibliotherapy were perceived as the least credible.

In terms of appeal, a significant difference was found between the methods (*F*_6,101_=15.3, *P*<.001, partial *η*^2^=0.48). Medication was perceived as the least appealing method across all options, while other methods were deemed equally appealing.

#### Cost, Privacy, and Waiting Time

A significant difference was found in the aspect of cost (F_6,104_=24.78, *P*<.001, partial *η*^2^=0.51). Face-to-face psychological intervention and medication were perceived as equally costly options compared to all other treatments and management methods. Guided internet-based psychological intervention performed better in fulfilling users’ expectations of cost as compared to face-to-face psychological intervention and medication but was perceived as more expensive than mobile apps.

Privacy and protection of personal information were significantly different between treatments and management methods (*F*_6,105_=6.01, *P*<.001, partial *η*^2^=0.043), with face-to-face psychological intervention and bibliotherapy or self-help books perceived as the best options for protecting privacy and personal information.

Waiting time was significantly different between treatments and management methods (*F*_6,103_=15.1, *P*<.001, partial *η*^2^=0.47). Mobile apps and bibliotherapy or self-help books were perceived to have the shortest waiting times, followed by guided internet-based psychological intervention. Face-to-face psychological intervention, medication, and counseling were perceived to be less satisfactory for their waiting times.

#### Ability to Improve Motivation and Flexibility in Service Provision

The ability to improve participants’ motivation to use depression treatments and management methods differed significantly (*F*_6,104_=12.1, *P*<.001, partial *η*^2^=0.41) too, with face-to-face psychological intervention, counseling, and psychological intervention via videoconferencing rated the highest for motivating users as compared to other management methods. Guided internet-based psychological intervention was perceived to be more able to improve users’ motivation than medication and bibliotherapy in this regard.

Perception of flexibility in service provision differed significantly (*F*_6,104_=11.5, *P*<.001, partial *η*^2^=0.40), with mental health apps and self-help literature rated most effective in providing flexible service times. Guided internet-based psychological intervention was found to better satisfy users’ expectations for flexible service times than face-to-face psychological intervention, medication, and counseling.

#### Location and Personalization

Location convenience differed significantly (*F*_6,104_=14.6, *P*<.001, partial *η*^2^=0.46), with internet-based psychological intervention, mental health apps, self-help literature, and psychological intervention via videoconferencing rated as the most convenient options. Face-to-face psychological intervention and medication were rated as the least convenient.

Expectation fulfillment related to personalization differed significantly across methods (*F*_6,106_=15.7, *P*<.001, partial *η*^2^=0.47), with face-to-face psychological intervention rated the highest. Internet-based psychological intervention was perceived as equally effective as medication and psychological intervention via videoconferencing in fulfilling potential service users’ expectations of personalization. Mental health apps and self-help literature were rated as the least effective in fulfilling users’ expectations of personalization.

#### Feedback Provision, Side Effects, and Timely Support

Feedback provision differed significantly (*F*_6,106_=30.5, *P*<.001, partial *η*^2^=0.63), with face-to-face psychological intervention rated the highest. Internet-based psychological intervention was equally effective as counseling and psychological intervention via videoconferencing in fulfilling potential service users’ expectations of feedback provision but was perceived to better provide feedback than mental health apps and self-help literature.

Concerns about side effects differed significantly (*F*_6,104_=30.4, *P*<.001, partial *η*^2^=0.63) across the studied depression management options, with medication rated the lowest in fulfilling potential users’ expectations.

Timely support differed significantly (*F*_6,106_=6.96, *P*<.001, partial *η*^2^=0.28), with face-to-face psychological intervention and self-help literature rated the worst in the provision of timely support.

#### Ability to Help Users Monitor Their Mental Health Status and Anonymity

The ability to help users to monitor their mental health status differed significantly (*F*_6,106_=26.1, *P*<.001, partial *η*^2^=0.60), with face-to-face psychological intervention rated as the most effective option, followed by guided internet-based psychological intervention, counseling, mental health apps, and psychological intervention via videoconferencing. Medication and self-help literature were rated as the least effective options.

Anonymity differed significantly (*F*_6,106_=7.80, *P*<.001, partial *η*^2^=0.60), with self-help literature rated as the most effective option. Guided internet-based psychological intervention was perceived to be more effective than face-to-face psychological intervention but not different from all other management methods with respect to anonymity.

**Figure 1:**
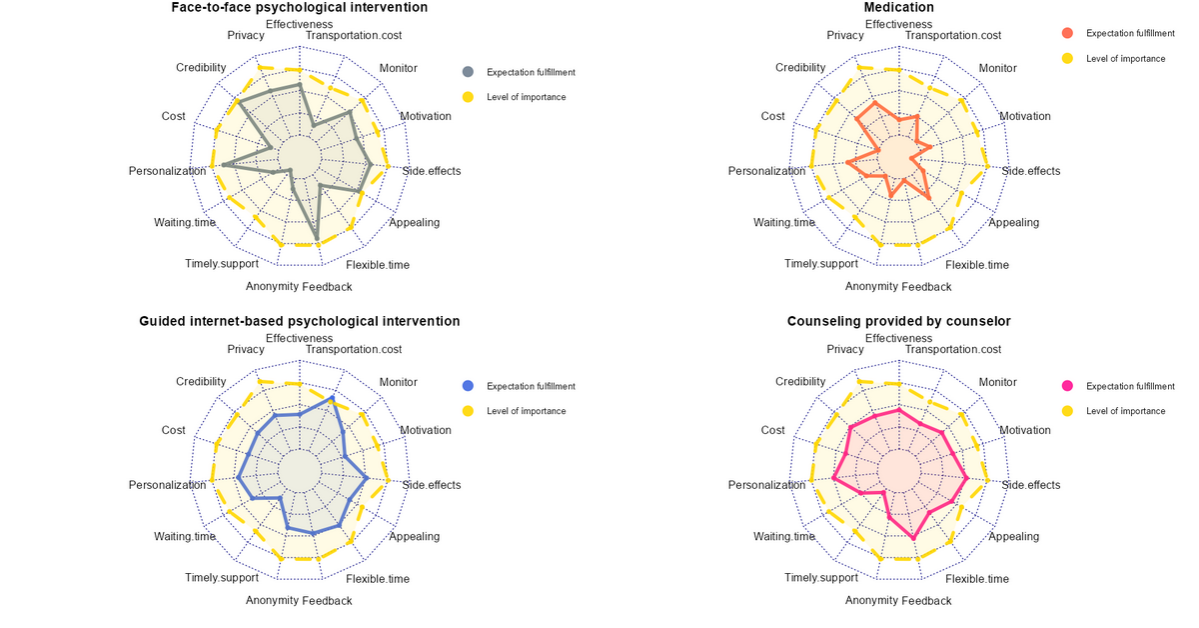
Radar charts of fulfilment of expectations across the 7 methods of managing depression.

**Figure 2:**
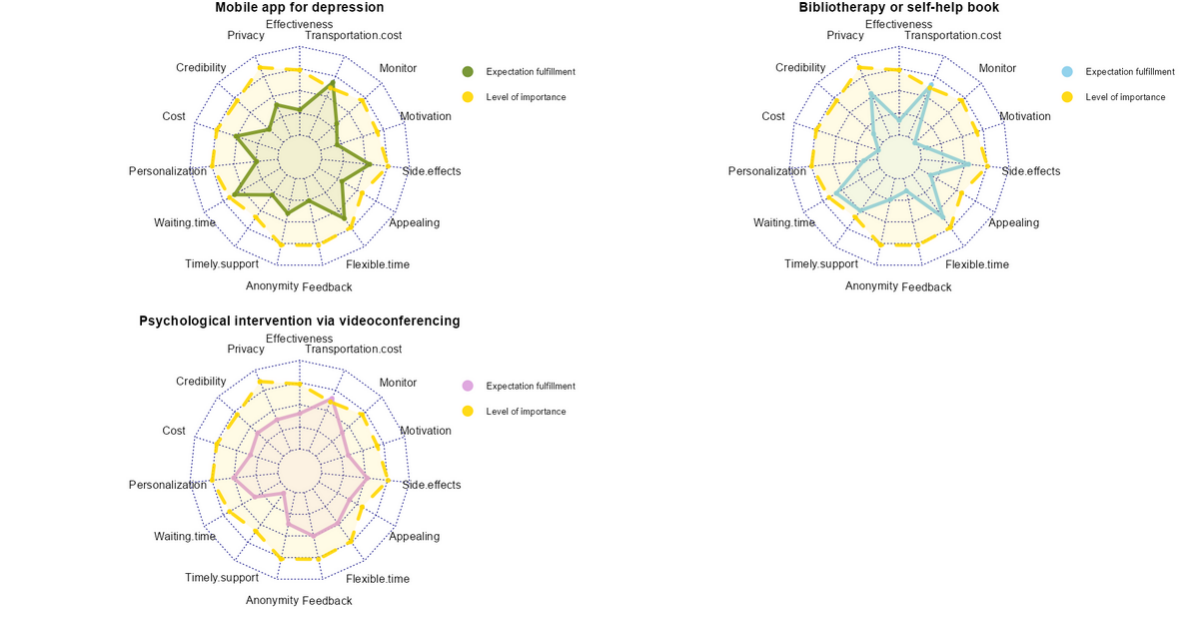
Radar charts of fulfilment of expectations across the 7 methods of managing depression.

Figures 1 and 2 illustrate the importance of each domain and participants’ ratings of how well each treatment fulfilled their expectations, providing insight into the strengths and weaknesses of each depression management method (see Table 4 for their mean scores).

**Table 4:** Mean scores of fulfilment of expectations across 7 methods of managing depression.

	Face-to-face psychological intervention, mean (SD)	Medication, mean (SD)	Guided internet-based psychological intervention, mean (SD)	Counseling provided by counselor, mean (SD)	Mobile app for depression, mean (SD)	Bibliotherapy or self-help book, mean (SD)	Psychological intervention via videoconferencing, mean (SD)
Effectiveness	5.28 (1.29)	3.68 (1.73)	4.56 (1.46)	4.76 (1.43)	4.13 (1.64)	3.65 (1.72)	4.6 (1.38)
Privacy	5.27 (1.58)	4.69 (2.02)	4.75 (1.52)	4.72 (1.51)	4.58 (1.65)	5.14 (1.68)	4.54 (1.56)
Credibility	5.71 (1.32)	4.58 (1.74)	4.56 (1.57)	4.95 (1.38)	3.86 (1.51)	3.56 (1.61)	4.57 (1.42)
Cost	3.38 (2.14)	2.99 (1.93)	4.44 (1.58)	4.55 (1.59)	5.04 (1.47)	4.91 (1.67)	4.35 (1.62)
Personalization	5.46 (1.57)	4.34 (1.94)	4.79 (1.45)	4.98 (1.51)	3.96 (1.75)	3.62 (1.73)	5.01 (1.43)
Waiting time	3.39 (1.89)	3.71 (1.8)	4.47 (1.54)	4 (1.61)	5.41 (1.44)	5.29 (1.68)	4.35 (1.46)
Anonymity	3.8 (2.11)	4.05 (2.15)	4.38 (1.65)	4.15 (1.85)	4.84 (1.63)	5.5 (1.62)	4.18 (1.73)
Timely support	3.48 (2.02)	3.79 (1.92)	4.62 (1.65)	4.14 (1.69)	4.61 (1.8)	3.97 (1.96)	4.45 (1.62)
Feedback provision	5.77 (0.12)	3.08 (0.17)	4.88 (0.14)	5.12 (0.13)	4.02 (0.15)	3.57 (0.16)	5.01 (0.13)
Flexible time in service provision	3.58 (1.93)	4.29 (1.82)	5.04 (1.45)	4.32 (1.51)	5.44 (1.39)	5.37 (1.48)	4.92 (1.52)
Appealing	5.11 (1.49)	3.25 (1.85)	4.6 (1.57)	4.74 (1.48)	4.21 (1.74)	3.64 (1.78)	4.6 (1.61)
Side effects	5.22 (1.58)	2.56 (1.9)	5.04 (1.57)	5.06 (1.5)	5.19 (1.54)	5.16 (1.65)	5.08 (1.53)
Being able to motivate service users to complete the service	4.7 (1.68)	3.47 (1.77)	4.15 (1.44)	4.55 (1.35)	3.78 (1.8)	3.33 (1.75)	4.31 (1.5)
Help users to monitor their mental health status	5.05 (1.6)	3.07 (1.78)	4.63 (1.51)	4.59 (1.56)	4.25 (1.81)	2.95 (1.65)	4.59 (1.47)
Transportation cost	3.56 (1.94)	4.01 (1.99)	5.63 (1.46)	4.34 (1.58)	5.69 (1.48)	5.57 (1.53)	5.59 (1.49)

### For Objective 3: To Compare the Likelihood of Using Different Management Methods for Depression

To understand the likelihood of use, participants indicated how likely they would use each of the 7 common mental health treatments and management methods. The result is summarized in Table 5. Repeated measures ANOVA was used to examine whether participants demonstrated the varied likelihood of use across each depression treatment and management method. The result was significant, with *F*_1,105_=10.94, partial *η*^2^=.39, *P*<.001. A pairwise comparison with a Bonferroni adjustment for multiple comparisons revealed that the mental health services could be summarized into 2 groups based on the likelihood of being used.

The first group, which had a higher likelihood of being used, included face-to-face psychological intervention, guided internet-based psychological intervention, counseling services provided by a counselor, and psychological intervention via videoconferencing. There were no significant differences (all *P*>.32) in the likelihood of use among these services.

The second group, which had a lower likelihood of being used, included bibliotherapy or self-help books, medication, and mobile apps for depression. There were also no significant differences (all *P*>.51) in the likelihood of use among these services.

**Table 5:** Likelihood of use for 7 methods of managing depression.

	Order by mean	Scores, mean (SE; range)
Face-to-face psychological intervention	1	4.36 (0.14; 4.08-4.64)
Guided internet-based psychological intervention	2	4.09 (0.13; 3.84-4.34)
Psychological intervention via videoconferencing	3	4.05 (0.12; 3.81-4.30)
Counseling provided by counselor	4	4.05 (0.13; 3.78-4.31)
Mobile app for depression	5	3.66 (0.14; 3.38-3.94)
Bibliotherapy or self-help book	6	3.51 (0.16; 3.20-3.81)
Medication	7	3.15 (0.16; 2.83-3.48)

## Discussion

### This Study

In this study, we investigated the importance of various psychological service attributes for individuals with depressive symptoms. In addition, the study examined the extent to which different treatment and management options fulfilled users’ expectations across 15 domains identified in the literature. The study also assessed the acceptability of these treatment options in terms of their likelihood of use.

### Principal Findings

#### Importance of Service Attributes Other Than Effectiveness

This study highlights the importance of considering various factors beyond the effectiveness of treatments when providing psychological services for individuals with depressive symptoms. Specifically, attributes such as privacy, feedback provision, anonymity, personalization, cost, flexible time in service provision, credibility, timely support and waiting time were all rated highly using an absolute rating of the importance of the service attribute. To the best of our knowledge, this study is the first to examine users’ valuation of service attributes in a sample of individuals above the clinical threshold for depression.

Our findings are consistent with previous research [19] that has highlighted the importance of various psychological service attributes in mental health treatment. For instance, previous studies have demonstrated service users’ concerns over undue disclosure, confidentiality, and privacy in mental health services [43-45] and in digital mental health services in particular [46]. Personalization and waiting time were also highly valued, highlighting the importance of tailored and individualized psychological interventions [47] and resolving implementational issues in the mental health care system [48,49]. Consistently, previous research has suggested that personalized mental-health interventions, personalization, and tailoring content toward users’ needs improved the user experience of psychological intervention [50], and that short waiting times did not only facilitate individuals with depressive symptoms to overcome barriers to seeking help [51]; recent evidence showed that the prolonged duration of the waiting time was associated with a less favorable treatment outcome [52].

While it may seem that users would prefer all positive attributes of a product or service, their preferences for these attributes can be revealed through ranking and trade-offs. Our study found that users rated effectiveness as the most important attribute among the 15 service attributes we studied, with privacy and protection of personal information, credibility, and cost following as the second most important batch of service attributes, following the most important attribute, “effectiveness.” These results are consistent with previous research that has highlighted the importance of these service attributes. For instance, a previous study found that users with mental health issues placed greater importance on scientific credibility over relational characteristics when considering psychological interventions for disorder-specific issues [53]. This underscores the importance of mental health service providers establishing and demonstrating scientific credibility and expertise in their field on top of building therapeutic relationships. Furthermore, the cost of services is an important factor to consider when providing mental health services. Previous findings suggest that the level of fees charged could have a significant impact on whether an individual would choose to seek therapeutic assistance, and financial barriers were one of the most often mentioned factors for not seeking treatment [54,55]. Therefore, both the research and the practice side of the health care system are recommended to put greater emphasis on the reduction of the cost of services and work to ensure psychological services are not only effective but also affordable and accessible to those who need them. These findings suggest that mental health service providers need to prioritize delivering effective treatments while also ensuring a myriad of factors, including protection of privacy and personal information, building credibility, and considering the cost of services when offering mental service options.

#### Assessing Acceptability: Considerations of Expectation Fulfillment and Likelihood of Use

The results of the study suggest that different depression management methods have strengths and weaknesses from the users’ perspective. For instance, face-to-face psychological intervention was rated as the most effective method for managing depression, while psychotropic medication and bibliotherapy or self-help books were rated as the least effective. Although bibliotherapy is a form of effective management method for depression as suggested by a recent meta-analysis [56], it is not surprising that potential users found it to be less effective than other management methods given its unguided nature. This finding is consistent with previous research that has shown that guided interventions, such as psychotherapy, are generally perceived to be more effective than unguided interventions, such as self-help books [57]. This finding informs researchers as well as clinicians of users’ possible holdbacks when being recommended this form of material.

Despite being viewed less favorably by users, it is worth noting self-help materials should not be less promoted or recommended, as they serve as low-cost, useful early interventions in primary care [58] and may be more accessible to individuals in certain contexts. For example, those who cannot afford or access traditional mental health services or those with lower e-literacy skills may find self-help literature more accessible. In addition, some participants in a study reported being more willing to engage with print media than with digital mental health services [59].

Notably, medication was rated as one of the least effective depression management methods which may be due to the perception that pharmacotherapy primarily addresses symptoms rather than underlying causes of mental health issues. This is consistent with previous research indicating that some service users perceive medication as “covering up the problem” rather than addressing it directly. Psychological intervention, by contrast, may be seen as a more preferred, logical, or effective approach with longer-lasting and broader effects [60].

In addition, it is important to note that users’ perceptions of the effectiveness of depression management methods may not always be updated with existing evidence. For example, this study found that participants rated guided internet-based psychological intervention as a less effective method for managing depression than face-to-face psychological intervention, despite numerous research suggesting the opposite [61]. This highlights the need for ongoing education and communication with users to ensure that they are aware of the evidence on the effectiveness of different depression management methods.

The study also found that guided internet-based psychological intervention was rated as more effective in fulfilling users’ expectations of cost compared to face-to-face psychological intervention and medication. This finding is consistent with previous research indicating that internet-based psychological intervention is a cost-effective alternative to traditional face-to-face psychotherapy [62,63]. As the inability to pay for services is frequently reported as a barrier to accessing mental health care [64], users’ perceptions of service costs are particularly important. In the era of managed care, where there is a heavy economic burden in meeting the needs of individuals who require mental health services [65], it is not sufficient for mental health services to only be effective; they must also be cost-effective and perceived as such by the public. Therefore, it is crucial to consider the cost-effectiveness of mental health services and how they are perceived by users to ensure the accessibility and sustainability of mental health care for all individuals in need. Regarding credibility, this study found that guided internet-based psychological intervention was perceived as less credible than face-to-face psychological intervention, which is consistent with previous research that has shown that service potential users may have concerns about the low credibility and impoverished communication between therapist and client in internet-based psychological intervention [25].

Practically, the findings of this study can inform the promotion and dissemination of depression management services. Providers of guided internet-based psychological intervention may consider emphasizing the low cost, short waiting time, and service flexibility of their services to improve the service appeal and uptake. It is also important to address the misconception that guided internet-based psychological intervention is a less effective and less credible alternative to face-to-face psychological intervention. By contrast, providers of face-to-face psychological intervention should recognize that their services may not meet some users’ expectations regarding waiting time and cost. However, their services are valuable to individuals who have more severe symptoms, have greater affordability, can tolerate long waiting times, or prefer in-person contact.

Furthermore, this study also serves as evidence that supports the continued development of digital health treatment services for people with elevated depressive symptoms, as the study sample showed that potential users were receptive to a wide range of services, as reflected by the fact that face-to-face psychological intervention, guided internet-based psychological intervention, counseling services provided by a counselor, and psychological intervention via videoconferencing all had an equal likelihood of being used. It contrasts with some of the previous findings [30] and could be due to contextual factors, such as the COVID-19 pandemic, which may have increased people’s willingness to try digital mental services. Offering and referring users to diverse service options and discussing the relative service strengths and weaknesses that users value are recommended and aligned with the latest National Institute for Health and Care Excellence guideline [66] that advocates shared decision-making, which is aimed at matching the choice to both clinical needs and personal preferences.

### Strengths and Limitations

The strength of this study is that this is the first study that attempted to understand how people with elevated depressive symptoms rate and prioritize various service attributes in depression treatment and management methods. This study is one of the few studies that examined how each depression treatment and management method fulfills users’ expectations on specific attributes. As compared to previous studies on the acceptance of digital mental health treatment services (eg, [19,67]), this study included both digital and nondigital options. Moreover, unlike previous studies that used a general population sample [19], this study specifically used a sample of individuals with depressive symptoms above the clinical threshold. This approach was chosen to enhance the validity and relevance of the study to real-world practice. Moreover, acceptability was investigated for various service attributes that were identified by service users as important aspects of mental health treatment. This method is preferred as it facilitates a thorough understanding of how a particular management method may or may not fulfill specific aspects of user expectations. It also provides insight into the specific strengths and weaknesses of the depression treatment and management methods.

Nevertheless, this study has several limitations. First, this study was conducted on the web, which may bias the result by sampling people who may have higher e-literacy. Future studies may aim to recruit participants through more diverse means of recruitment channels to enhance the generalizability of the findings. Second, as in other studies targeting public acceptability toward digital mental health [67], we used a self-developed survey that was not psychometrically validated. Although the service attributes investigated in this study were based on previous research on factors that influence users’ decisions to engage in a particular treatment option for mental health problems [19], it is possible that we may have missed out on some important service attributes, or some service attributes may not be psychometrically distinguishable. Although a validated scale on digital mental health service acceptance [68] exists, to the best of our knowledge, there is currently no validated scale on mental health service acceptance that specifically covers how people perceive it as fulfilling their expectations of various important service attributes commonly raised by users, such as waiting time, cost, and more. The development and validation of such a scale would be valuable for future studies, as it would enable a more comprehensive and standardized approach to exploring and measuring acceptance in both digital and nondigital mental health services. Finally, the acceptability in this study focused on the users’ perception and behavioral intention; although these may serve as important factors contributing to the initiation of treatments for depression [69], acceptability could also be conceptualized beyond the attitudinal level and manifested in behavior, such as actual uptake and continuation of use of treatments [70]. Yet, the behavioral aspect of acceptance, including adherence and dropout rates, could not be investigated in this study. It would be valuable for future research to explore and compare the behavioral aspects of acceptance across different treatments for depression or management methods in the future. This could involve synthesizing previous studies and investigating factors that contribute to treatment adherence, exploring reasons for treatment discontinuation, and comparing postservice satisfaction [71] of the various digital and nondigital approaches to managing depression.

### Conclusions and Implications

This study provided valuable insights into the users’ perspectives on various psychological treatments for depressive symptoms. It assessed the acceptability of these management methods using 15 service attributes and stated the likelihood of use. The findings indicated that individuals with elevated depressive symptoms are equally likely to consider adopting guided internet-based psychological intervention as they would be to consider the face-to-face version. This study also highlighted the specific strengths and weaknesses of different treatment and management methods for depressive symptoms. It is important to consider these factors when offering treatment options to individuals. Furthermore, because acceptance of health services could potentially be changed by social marketing [72] and aids in decision-making [35,73], efforts should continue to be made to disseminate credible and accurate information to the public, ensuring that people have access to comprehensive information about different treatment options, especially on service attributes that they valued. This would enable individuals to make informed choices regarding their mental health treatment and management.

## Abbreviations

PHQ: Patient Health Questionnaire

## Data Availability

The dataset analyzed in this study are available from the corresponding author upon reasonable request. These data are not publicly available, as they contain information that could compromise the privacy of the research participants.
